# Histamine and TNF-α release by rat peritoneal mast cells stimulated with *Trichomonas vaginalis*

**DOI:** 10.1051/parasite/2011181049

**Published:** 2011-02-15

**Authors:** S.J. Im, M.H. Ahn, I.H. Han, H.O. Song, Y.S. Kim, H.M. Kim, J.S. Ryu

**Affiliations:** 1 Department of Environmental Biology and Medical Parasitology, Hanyang University College of Medicine #17 Haengdang-dong Sungdong-gu, Seoul 133-791 Korea; 2 Department of Parasitology, School of Medicine, Catholic University of Daegu Daegu 705-718 Korea; 3 Department of Biochemistry and Molecular Biology, Hanyang University College of Medicine Seoul 133-791 Korea; 4 Department of Pharmacology, College of Oriental Medicine, Institute of Oriental Medicine, Kyung Hee University Seoul 130-701 Korea

**Keywords:** *Trichomonas vaginalis*, rat, mast cell, migration, histamine, TNF-α, *Trichomonas vaginalis*, rat, mastocyte, migration, histamine, TNF-α

## Abstract

Mast cells have been reported to be predominant in the vaginal smears of patients infected with *T. vaginalis*. In this study, we investigated whether *T. vaginalis* could induce mast cells to migrate and to produce TNF-α and histamine. Rat peritoneal mast cells (RPMC), a primary mast cell, were used for the study. *T. vaginalis* induced an increase in chemotactic migration of the mast cells toward excretory and secretory product (ESP) of *T. vaginalis*, and the mast cells activated with *T. vaginalis* showed an increased release of histamine and TNF-α. Therefore, mast cells may be involved in the inflammatory response caused by *T. vaginalis*.

## Abstract (fr)

Les mastocytes sont observés en quantité importante dans les sécrétions vaginales de patientes infectées par *T. vaginalis*. Cette étude explore pourquoi *T. vaginalis* induit la migration des mastocytes et leur production de TNF-α et d’histamine. Des mastocytes péritonéaux du rat ont été utilisés pour cette étude. Les produits d’excrétion-sécrétion de *T. vaginalis* induisent une augmentation de la migration chémotactique des mastocytes, et les mastocytes ainsi activés par *T. vaginalis* augmentent leur production d’histamine et de TNF-α. Les mastocytes seraient donc impliqués dans la réaction inflammatoire à *T. vaginalis*.

## Introduction

*Trichomonas vaginalis* is the most common curable sexually-transmitted infection (STI) worldwide (Johnston & Mabey, 2008). In pregnant women, trichomonads are implicated in the premature rupture of membranes, premature delivery, and the delivery of low-birth weight infants (Cotch *et al.*, 1997; Moodley *et al.*, 2002). In addition, trichomoniasis has been implicated as a risk factor for human immunodeficiency virus transmission (McClelland et al., 2007). More than 174 million people worldwide are infected by this parasite annually, and the prevalence rate was recently found to be 10.4% in the area of Guri, Korea (Johnston & Mabey, 2008; Ryu & Min, 2006).

Mast cells have been implicated as effector cells in response to multiple nematodes and other parasitic organisms. Specific mast cell proteases such as mast cell protease (MCP-9 and MCP-1) have been shown to have crucial roles in mediating responses to nematode infection. (McDermott et al., 2003; Lawrence *et al.*, 2004). However, their role in protozoan infections is less well described (Li *et al.*, 2004). In trichomoniasis, it has been reported that an increased frequency of mast cells are commonly found in the vaginal smears and vaginal walls of infected women (Kobayashi *et al.*, 1983; Karagiosov & Pophristova, 1978; Dawicki & Marshall, 2007). Although these observations suggest that mast cells may be involved in the cellular reaction to vaginal trichomoniasis, mast cell infiltration and their role in immunity against trichomoniasis has not yet been clearly characterized.

Mast cells are strategically located at sites that interface with the external environment, and are closely associated with blood vessels and nerves. As longlived cells, they can have an enormous impact on the tissue microenvironment through the selective release of a wide variety of preformed and newly derived mediators including potent proteases, cytokines, chemokines, and arachidonic acid metabolites (Dawicki & Marshall, 2007). Among these immunomodulatory molecules, TNF-α and histamine are prototype mediators secreted by activated mast cells and induce an inflammatory reaction.

However, to date, no reports have described TNF-α or histamine production by mast cells after stimulation with *T. vaginalis*. In this study, to elucidate the involvement of mast cells in *T. vaginalis* infection, we examined TNF-α and histamine production in rat peritoneal mast cells activated by *T. vaginalis* and measured the chemotactic migration of mast cells towards *T. vaginalis*.

## Materials and Methods

### Reagents

PMA, calcium ionophore A23187, percoll, and heptane were purchased from Sigma Chemical Co. (St. Louis, MO, USA). Fetal bovine serum (FCS) and horse serum were purchased from Gibco BRL (Gaithersburg, MD). Recombinant rat stem cell factor was purchased from PeproTech Asia (Israel), and *o*-phthaldialdehyde was purchased from Biochemica (Austria).

### Cell cultures

The *Trichomonas vaginalis* isolate T016 used in the present study was grown in a complex trypticase-yeast extract-maltose (TYM) medium supplemented with 10% heat-inactivated horse serum. The excretorysecretory products (ESP) of *T. vaginalis* were obtained by suspending trophozoites (1 x 10^7^/ml) in RPMI 1640, culturing them at 37 °C for 1 h, and then centrifuging at 10,000 *g* for 30 min. The resulting supernatants were passed through a 0.22-μm filter. Undiluted ESP called 100% ESP. Phosphate buffered saline (PBS) was used for dilution of the 100% ESP, and percent of ESP depends on the amount of PBS.

### Animals

Wistar rats were purchased from Samtako (Kyung Gi-Do, Korea). They were housed 5-10 per cage in a laminar air-flow room maintained at a temperature of 22 ± 4 °C and relative humidity of 55 ± 5% throughout the study.

### Rat peritoneal mast cell (RPMC) preparation

RPMCs were isolated as described previously (Kim & Lee, 1999). In brief, Wistar rats were anesthetized by ether and injected with 50 ml of Tyrode buffer (137 mM NaCl, 5.6 mM glucose, 12 mM NaHCO_3_, 2.7 mM KCl, 0.3 mM NaH_2_PO_4_, and 0.1% gelatin) into the peritoneal cavity, and the abdomen was gently massaged for about 90 s. The peritoneal cavity was carefully opened, and the fluid containing peritoneal cells were aspirated by Pasteur pipette. The peritoneal cells were sedimented at 150 g for 10 min at 4 °C and resuspended in Tyrode buffer. Mast cells were separated from the major components of rat peritoneal cells, *i.e.* macrophages and small lymphocytes, according to the method described by Yurt *et al.* (1977). In brief, peritoneal cells were suspended in 1 ml of Tyrode buffer, layered on 2 ml of 45% percoll (density 1.12) and centrifuged at 4 °C for 15 min at 400 *g*. The cells remaining at the buffer-percoll interface were aspirated and discarded; the cells in the pellet were washed and resuspended in 1 ml of Tyrode buffer. Mast cell preparations were approximately 96% pure as assessed by toluidine blue staining. More than 97% of the cells were viable as measured by trypan blue uptake.

### Transmission electron microscopy

Transmission electron microscopy was conducted as follows: RPMC (3 × 10^6^) were reacted with live trichomonads (1.5 × 10^6^) or *T. vaginalis* ESP (25%) incubated for 3 h, fixed with 3% glutaraldehyde in 0.1 M phosphate buffer (pH 7.3) for 3 h, washed three times, and postfixed with 1% osmium tetroxide in 0.1 M phosphate buffer for 2 h. The specimens were then dehydrated in an ethanol gradient, embedded in Epon, and polymerized at 60 °C for 48 h. Ultra-thin sections (50-70 nm) were stained with 1% uranyl acetate followed by lead citrate, and examined with a transmission electron microscope (H-7600S, Hitachi, Japan) (Kang *et al.*, 2006).

### Assay of histamine release

Histamine content was measured by the *o*-phthaldialdehyde spectrofluorometric procedure (Shore *et al.*, 1959; Schwartz *et al.*, 1981; Na *et al.*, 2005). RPMC suspensions (2 × 10^5^) were reacted with *T. vaginalis* ESP (10%, 25%, 50%), and live trichomonads (1 × 10^5^, 4 × 10^5^) for 30 min. The reaction was stopped by cooling the tubes on ice. The cells were separated from the released histamine by centrifugation at 400 *g* for 5 min at 4 °C. Residual histamine in the cells was released by disrupting the cells with 1% Triton-X 100 and centrifuged at 400 *g* for 5 min at 4 °C. In brief, the culture supernatant (250 μl each) were mixed with 225 μl of 0.1 N HCl and 25 μl of 60% perchloric acid and centrifuged 22,000 *g* at 4 °C for 20 min. The collected supernatant (400 μl), water (1.5 ml), and *n*-butanol (5 ml) were put into a tube containing NaCl (0.6 g) and 5 N NaOH (250 μl), and mixed for 20 min and centrifuged at 385 *g* for 5 min at 4 °C. The collected supernatant (4 ml) and heptane (5 ml) were put into a tube containing 1.5 ml of 0.1 N HCl, and mixed for 20 min and centrifuged as above. The liquid collected from bottom level (150 μl), 1 N NaOH (30 μl), and 7.5 μl of 1% *o*-phthaldialdehyde (OPT) were put into optiplate 96-well plates (Costar) and incubated for 3 min at 30 °C. To stop the reaction, 3 N HCl (15 μl) was added and the light emission was measured in a spectrofluorometer. The fluorescent intensity was measured at 440 nm (excitation at 360 nm) in a spectrofluorometer (Varioskan Flash, Thermo Electron Corporation, Mass, USA). Histamine release was calculated as a percentage (released histamine) of total histamine (= extracellular plus intracellular histamine).

### TNF-α expression

TNF-α protein in supernatants from RPMCs (2 × 10^5^) stimulated with live trichomonads (5 × 10^5^, 10 × 10^5^) or *T. vaginalis* ESP (10%, 25%, 50%) was measured by ELISA using a commercial kit (BD Biosciences) according to the manufacturer’s instructions.

MRNA expressions of TNF-α was measured by RTPCR after RPMC (1 × 10^6^) coincubated with 50% ESP of *T. vaginalis* for 1 h or 2 h. Total RNA was extracted from cells using Trizol reagent (Invitrogen, Carlsbad, California, USA) according to the method described previously (Han *et al.*, 2009). Primer sequences and PCR conditions used for amplification of β-actin and TNF-α were as follows: β-actin (sense) 5’-AGCCATGTACGTAGCCATCC-3’, (antisense) 5’-CTCTCAGCTGTGGTGGTGAA-3’; TNF-α (sense) 5’-CTCTTCAAGGGACAAGGCTG-3’, (antisense) 5’- TGGAAGACTCCTCCCAGGTA-3’; PCR conditions of initial DNA denaturation at 94 °C for 5 min and 35 rounds of denaturation (98 °C for 15 sec), annealing (53 °C for 30 sec), and extension (72 °C for 35 sec). The PCR products were electrophoresed in 2% agarose gel containing 0.5 μl/ml ethidium bromide, and photographed under ultraviolet light. The band intensity was quantified using the Quantity One program (BioRad, Hercules, California, USA) (Han *et al.*, 2009).

### Chemotaxis assay

To test migration of the mast cells, a cell migration assay was performed using a 24-well microplate. The lower wells were filled with 500 μl buffer containing *T. vaginalis* ESP (10%, 25%, 50%) or recombinant rat SCF (100 ng/ml). A polyvinylpyrrolidone-free polycarbonate filter (Millipore, Ireland) with an 8 μl pore-size was then placed over the lower well. The upper wells were filled with 200 μl of RPMCs (2 × 10^5^ total cells) in MEM containing 10% fetal bovine serum. The plate was incubated for 4 h at 37 °C. After the filter was removed, the cells adhering to its upper surface were wiped off with a filter wiper. The filter was dried, fixed, and stained with 0.5% toluidine blue. The cells from four randomly selected fields per well were counted using a light microscope. The chemotactic index was calculated by comparing the number of cells that had migrated in the experimental sample to the number of cells that had migrated in the control.

### Statistical analysis

The results are expressed as means ± SEM of three to four independent experiments. The Mann-Whitney *U*-test was used for statistical analysis, and a P value of < 0.05 was considered statistically significant.

## Results

### Electron microscopic features of RPMC

**Fig 1.**
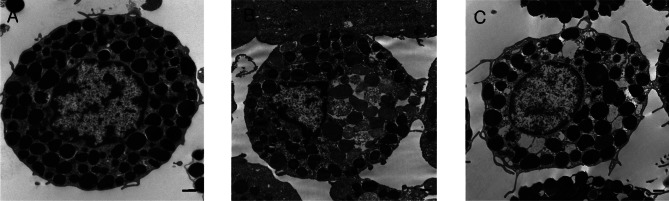
Electron microscopy of rat peritoneal mast cells (RPMC). (A) untreated RPMC, (B) & (C) RPMC treated with live trichomonads and 25% ESP of *T. vaginalis* for 3 h, respectively. Typical mast cell-specific granules (granules filled with electron-dense material) are present in untreated RPMC, which were presumably exocytosed after stimulation by live trichomonad or *T. vaginalis* ESP. Scale bar: 0.5 μm.

Freshly isolated RPMCs contained many spherical electron-dense granules in the cytoplasm. Mast cell treated with live trichomonads or *T. vaginalis* ESP showed decreased number of electron-dense granules. *T. vaginalis* ESP caused loss of a few granule from RPMC. In case of RPMC treated with live trichomonads, several granules were swollen and less electron dense. The presented data suggest that the *T. vaginalis* induces the exocytosis of mast cell-specific granules (Fig. 1).

### Histamine release

**Fig 2.**
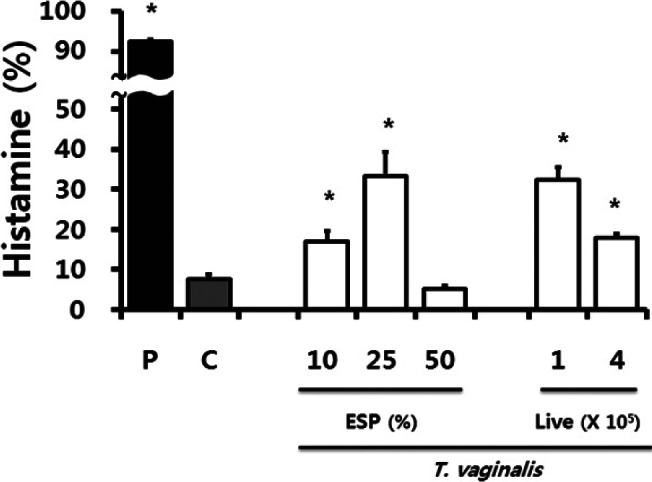
Histamine release from rat peritoneal mast cells stimulated with *T. vaginalis* (excretory-secretory products of *T. vaginalis*, and live trichomonads) for 30 min. P: PMA (50 nM) + A23187 (1 μM); C: RPMC alone. * p < 0.05 *versus* RPMC alone.

Among the preformed and newly synthesized inflammatory substances released upon the degranulation of mast cells, histamine remains the best-characterized and most potent vasoactive mediator implicated in the acute phase of anaphylaxis (Petersen *et al.*, 1996). When RPMCs were stimulated with *T. vaginalis* ESP (10%, 25%, 50%), or live trichomonads (1 × 10^5^, 4 × 10^5^) for 30 min, the mast cells showed a significant increase in histamine release compared with untreated RPMCs except of that of 50% ESP with decreased level. As expected, the combination of PMA and A23187, used as a positive control, significantly increased histamine release by RPMCs to nine times more compared with that of medium alone (Fig. 2).

### TNF-α production

**Fig 3.**
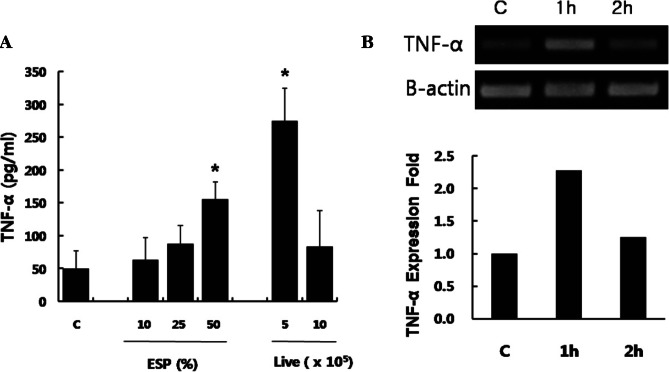
(A). TNF-α production induced by rat peritoneal mast cells stimulated with *T. vaginalis* (excretory-secretory products of *T. vaginalis*, and live trichomonads) for 6 h. * p < 0.05 *versus* RPMC alone. C: RPMC alone. (B) TNF-α mRNA expression of RPMC stimulated with 50% ESP of *T. vaginalis* for 1 h or 2.

Activated mast cells produce the multipotent cytokine TNF-α (Gordon & Galli, 1991). *T. vaginalis* ESP and live trichomonads induced an increase in TNF-α production by RPMCs compared with that of medium alone, while live trichomonads induced the highest level of production. When RPMCs were stimulated with live trichomonads for 60 min, TNF-α mRNA expression increased by two-fold compared with medium alone (Fig. 3).

### Chemotaxis assay

**Fig 4.**
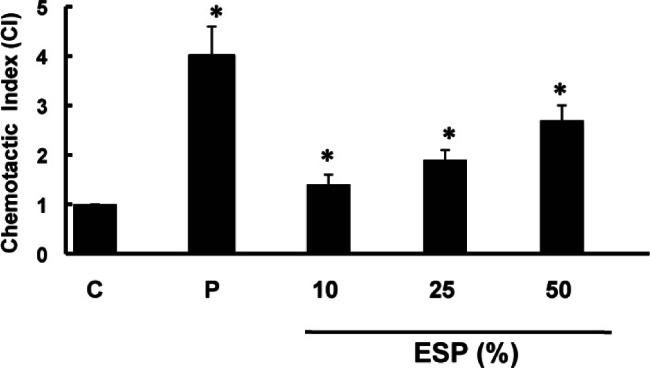
The migration of rat peritoneal mast cells was assessed by counting the number of RPMCs which migrated through the polycarbonate membrane after treatment with the excretory-secretory products (ESP) of *T. vaginalis* for 4 h. Stem cell factor (SCF) at 100 ng/ml was used as a positive control (P). C: RPMC alone. * p < 0.05 *versus* RPMC alone.

Because cell migration is a pivotal step in the inflammatory response, we investigated the alteration of RPMC migration after stimulation with *T. vaginalis*. *T. vaginalis* ESP induced increased migration of RPMCs, and was maximally active at a concentration of 50%. As expected, stem cell factor (SCF), used as positive control, increased the chemoattraction of RPMCs (Fig. 4).

## Discussion

In trichomoniasis, vaginal discharge and vulvovaginal irritation are known to be the most common presenting complaints. The vulvovaginal pruritus or irritation is noted by one fourth to four fifths of infected women (Rein, 1989). Additionally, the vaginal walls are recognized as erythematous in perhaps one-third to two-thirds of patients (Gardner, 1981). Increased vascularity and a characteristic vascular pattern in the squamous epithelium of the cervix as well as an increased abundance of mast cells in the endocervical smear were described in *T. vaginalis* cervicitis (Kobayashi *et al.*, 1983; Kolstad, 1964; Bergsjoe *et al.*, 1963; Skrzypiec, 1977). These main signs and symptoms suggest that *T. vaginalis* stimulates an allergic reaction. Furthermore, relatively high IgE levels have been measured in patients with vaginal trichomoniasis and in mice immunized with a soluble antigen of *T. vaginalis* (Yano *et al.*, 1982; Green *et al.*, 1976). Although these observations suggest that mast cells may be involved in the allergic and cellular reaction to vaginal trichomoniasis, involvement of mast cell didn’t experimentally proved. From this study, *T. vaginalis* induced an increase in chemotactic migration of the mast cells toward *T. vaginalis*, and the mast cells activated with *T. vaginalis* showed an increased release of histamine and TNF-α. These results provide experimental confirmation of mast cell involvement in *T. vaginalis* infection.

*Giardia lamblis* is similar to *T. vaginalis* in that they are both flagellate protozoans and extracellular parasites in humans. Mast cell responses may contribute to the pathophysiology of giardiasis in addition to the control of infections. Symptoms of giardiasis include malabsorptive diarrhea, cramps, and nausea and are similar to those noted in food allergies, celiac disease, and other intestinal disorders. In several of these diseases and in several animal models, mast cells have been implicated in contributing to this pathology (Farthing, 2003). Additionally, the reported increase in serum MCP-1 and histamine levels observed after infection of C57BL/6 mice suggest that symptoms of giardiasis may be related to allergic processes (Di Prisco *et al.*, 1998).

A particular problem of human mast cell research is the difficulty in obtaining human cell material for in vitro studies. Therefore, most in vitro mast cell experiments have been carried out either with human cell materials that might have limited functional significance, such as transformed cell lines or partially immature mast cells, or with murine primary mast cells that can be easily obtained, such as peritoneal mast cells. Murine peritoneal cells have been widely used, as a single peritoneal lavage yields large numbers of mast cells that can be easily purified (Bischoff, 2007). Therefore, rat peritoneal mast cells, a primary mast cell, were used for this study

In the present study, rat peritoneal mast cells demonstrated increased migration toward *T. vaginalis* ESP. Interestingly, we and other researchers previously described a chemotactic activity of *T. vaginalis* ESP on leukocytes (Mason & Forman, 1982; Park *et al.*, 1984). This suggests that *T. vaginalis* ESP may induce the migration of mast cells as well as leukocytes. Although the exact component of *T. vaginalis* ESP, which is chemoattractive is not known, Leukotriene B4 (LTB4) in ESP was reported to play a major role in chemotactic activity (Shaio *et al.*, 1992). Further experiment will be examined whether anti-LTB4 antibody could abolish the chemotactic activity of ESP.

When mast cells are activated, they immediately extrude granule-associated substances such as histamine, and within minutes generate lipid-derived mediators (Brody & Metcalfe, 1998) In addition, mast cell activation is followed within hours by the *de novo* synthesis of numerous cytokines and chemokines (Kobayashi *et al.*, 2000). The production of TNF-α and histamine in *T. vaginalis*-stimulated mast cells were increased in dose-dependent manner except those of highest concentrations. In further study, application of various incubation periods, including shorter incubation time, for histamine or TNF-α production would be required to find reason of the decreased level on highest concentration of parasites.

It has been shown that mast cells, and mast cellderived TNF, can promote the migration of other hematopoietic cell types, including neutrophils, monocytes, and T cells, in the context of both innate and acquired immune responses (Suto *et al.*, 2006; Malaviya *et al.*, 1996; Biedermann *et al.*, 2000). In addition, histamine and TNF produced by IgE- and Ag-activated mast cells have been shown to be important for the migration of subsets of DCs from the skin and airways to the lymph nodes (Gordon & Galli, 1991; Suto *et al.*, 2006; Jawdat *et al.*, 2004). The production of TNF-α and histamine in *T. vaginalis*-stimulated mast cells were shown to increase in this study. Secreted mediators including TNF-α and histamine are expected to aid in the recruitment of neutrophils, and to initiate the subsequent adaptive immune response. Therefore, further studies need to be carried out to investigate cell migration of other hemopoietic cell types stimulated by *T. vaginalis*.

Therefore, *T. vaginalis* induced an increase in chemotactic migration of the mast cells toward *T. vaginalis*, and the mast cells activated with *T. vaginalis* showed an increased release of histamine and TNF-α. In conclusion, it is suggested that mast cells play a role in the inflammation caused by *T. vaginalis* by producing histamine and cytokines.
